# Decoding imagined speech with delay differential analysis

**DOI:** 10.3389/fnhum.2024.1398065

**Published:** 2024-05-17

**Authors:** Vinícius Rezende Carvalho, Eduardo Mazoni Andrade Marçal Mendes, Aria Fallah, Terrence J. Sejnowski, Lindy Comstock, Claudia Lainscsek

**Affiliations:** ^1^RITMO Centre for Interdisciplinary Studies in Rhythm, Time and Motion, University of Oslo, Oslo, Norway; ^2^Postgraduate Program in Electrical Engineering, Universidade Federal de Minas Gerais, Belo Horizonte, MG, Brazil; ^3^Department of Neurosurgery, University of California, Los Angeles, Los Angeles, CA, United States; ^4^Computational Neurobiology Laboratory, The Salk Institute for Biological Studies, La Jolla, CA, United States; ^5^Institute for Neural Computation University of California, San Diego, La Jolla, CA, United States; ^6^Department of Neurobiology, University of California, San Diego, La Jolla, CA, United States; ^7^Department of Psychiatry and Biobehavioral Sciences, Semel Institute for Neuroscience and Human Behavior, University of California, Los Angeles, Los Angeles, CA, United States

**Keywords:** delay differential analysis, non-linear dynamics, signal processing, speech decoding, electroencephalography

## Abstract

Speech decoding from non-invasive EEG signals can achieve relatively high accuracy (70–80%) for strictly delimited classification tasks, but for more complex tasks non-invasive speech decoding typically yields a 20–50% classification accuracy. However, decoder generalization, or how well algorithms perform objectively across datasets, is complicated by the small size and heterogeneity of existing EEG datasets. Furthermore, the limited availability of open access code hampers a comparison between methods. This study explores the application of a novel non-linear method for signal processing, delay differential analysis (DDA), to speech decoding. We provide a systematic evaluation of its performance on two public imagined speech decoding datasets relative to all publicly available deep learning methods. The results support DDA as a compelling alternative or complementary approach to deep learning methods for speech decoding. DDA is a fast and efficient time-domain open-source method that fits data using only few strong features and does not require extensive preprocessing.

## 1 Introduction

The field of speech decoding has made significant advances in recent years, yet current technologies still suffer from major limitations in speed, accuracy, and practicality: brain-computer interface (BCI) devices that utilize neural signals from invasive implants remain inaccessible for the majority of their target user population, and devices that decode from non-invasive electroencephalography (EEG) signals remain insufficiently fast with low accuracy and a limited range of classification abilities.

The use of scalp electroencephalography (EEG) as neural inputs for speech decoding is a non-invasive and affordable method that can be made widely available for both clinical and non-clinical populations. Nonetheless, the sometimes poor quality of EEG signals considerably limits the complexity of its applications; therefore, careful thought is required to determine the best means to develop future real-world applications (Lopez-Bernal et al., 2022). Different brain-computer interface (BCI) paradigms have evolved in the last decade for decoding from non-invasive EEG signals (Abiri et al., 2019). Motor imagery paradigms are often employed, given the robust nature of motor signals. Motor circuits play an integral role in speech production and comprehension via the neuromotor commands that are sent to muscles controlling speech articulation (Liberman et al., 1967; Comstock et al., 2019).

Imagined speech paradigms will activate neuromotor signals, although the signals may be less robust than when words or sounds are mouthed or spoken. These paradigms are characterized by a participant imagining the articulatory movements involved in the generation of different target phonemes and words (Panachakel and Ramakrishnan, 2021), which capitalizes on the fact that imagining a movement will still lead to activation of the areas of the brain involved in generating movements. An even more subtle signal is generated by inner speech (also called internal speech or covert self-talk), which involves participants thinking about specific words, but without reconstructing the required articulation to generate them. This approach offers additional challenges for decoding due to the more complex and distributed nature of the underlying neural activity (Nieto et al., 2022). Nonetheless, imagined speech paradigms have proved popular, because they are argued to represent the most naturalistic data that might be easily produced by patients with locked-in syndrome (Cooney et al., 2022).

EEG-based BCI devices currently involve paradigms that allow for the selection of one item out of a set of commands or sounds but are constrained in the scope and content of that set, increasing the chance of successful decoding; when fewer options are involved, the complexity of the decoding task is decreased. That is, using fewer classes for decoding typically simplifies acoustic and language modeling, while also reducing ambiguity and computational requirements. Most imagined speech EEG datasets contain relatively few classes, consisting of command words (Alejandro Antonio Torres Garcia, 2012; Nguyen et al., 2018; García-Salinas et al., 2019; Pawar and Dhage, 2023), vowels (/a/, /e/, /i/, /o/, /u/) (Matsumoto and Hori, 2014; Min et al., 2016; Pressel Coretto et al., 2017), or phonemes composed of different articulatory features (Brigham and Kumar, 2010; Deng et al., 2010; Zhao and Rudzicz, 2015). Naturalistic approaches are being sought, but face challenges regarding within-subject inter-section and inter-subject variabilities (Sharon et al., 2020).

The rapid expansion of deep-learning and signal processing methods has led to promising state-of-the-art speech decoding methods, with results that go far beyond near-chance level findings (Pressel Coretto et al., 2017), as shown in Tables 1, 2 (for detailed reviews, see Panachakel and Ramakrishnan, 2021; Lopez-Bernal et al., 2022; Shah et al., 2022). However, comparing between these methods and assessing how generalizable they are is currently a difficult task because few papers make their data or code publicly available (Shah et al., 2022). Furthermore, the datasets possess a limited number of trials, participants, and classes, making it difficult to assess their real efficacy. There is still a lack of well-established benchmarks, and the reported evaluation metrics are limited to accuracy (percent correct) in most cases (Shah et al., 2022), which is an inadequate criterion for some datasets.

**Table 1:** Performance metrics for UNL-CONICET dataset (Pressel Coretto et al., 2017).

**Work**	**Method**	**CV**	**SD**	**Problem**	**Accuracy**
Pressel Coretto et al. (2017)	DWT + SVM/RF	Train S1-S3, test on S4-S15	No	Vowels	22%
				Words	19%
Cooney et al. (2019)	CNN	5-fold	Yes	Vowels	33%
	CNN + TL	5-fold + LOO	No		36%
Cooney et al. (2020)	CNN/DL	Nested CV with HP optimization	Yes	Vowels	30%
				Words	24%
			No	Vowels	30%
				Words	25%
Simistira Liwicki et al. (2022)	CNN	LOO	Yes	Vowels	35%
				Words	29%
Sarmiento et al. (2021)	CNNeeg1-1	70% train/30% test	Yes	Vowels	66%
Tamm et al. (2020)	CNN	5-fold CV	Yes	Vowels	24%
García-Salinas et al. (2018)	PARAFAC + SVM	10-fold CV (S01-S03)	Yes	Vowels	60%
Lee et al. (2020)	Siamese NN	5-fold CV	Yes	Words	31%
Lee et al. (2021)	Deep metric learning	5-fold CV	Yes	Words	45%
Biswas and Sinha (2022)	DWT + CSP	5-fold CV	Yes	Words	41%
Current study	DE-DDA + SVM	DE-DDA + 5-fold CV	Yes	Vowels	37%
				Words	34%
	DE-DDA + SVM	LOO	No	Vowels	19%
				Words	17%

CSP, common spatial pattern; DWT, discrete wavelet transform; TL, transfer learning; PARAFAC, parallel factorial analysis; LOO, leave one out.

**Table 2:** Performance metrics for Kara One dataset (Zhao and Rudzicz, 2015).

**Work**	**Method**	**Training/CV**	**SD**	**Problem**	**Accuracy**	**AUC**	**PPV**	**Recall**	**F1 score**
Zhao and Rudzicz (2015)	Statistical features + DBN/SVM	LOO	No	C/V	87				
				Nasal	63				
				Bilabial	57				
				iy	60				
				uw	82				
Bakhshali et al. (2020)	CSD Riemannian distance + KNN	80% train/20% test	Yes	C/V	87				
				Nasal	72				
				Bilabial	69				
				iy	75				
				uw	84				
Sun and Qin (2016)	NN+RBM	70% train/30% test	No	C/V	25				
				Nasal	47				
				Bilabial	53				
				iy	53				
				uw	74				
				11-class	42				
Saha et al. (2019)	CNN + deep autoencoder	80%/10%/10%	No	C/V	89		0.86	0.65	0.75
				Nasal	78		0.67	0.7	0.69
				Bilabial	82		0.72	0.76	0.47
				iy	87		0.86	0.78	0.82
				uw	85		0.77	0.57	0.65
				11-class	53				
Cooney et al. (2018)	MFCCs + SVM	5-fold CV	Yes	11-class	20				
Rusnac and Grigore (2022a)	CNN	4-fold CV	No	11-class	37			0.38	
Rusnac and Grigore (2022b)	LDA+ CNNLSTM	4-fold CV	No	11-class	44			0.44	
Mini et al. (2021)	SMRT/MFCC/LPCC + PCA/ANN	10-fold CV	Yes	11-class	77				
Bakhshali et al. (2022)	pCBCSD + SVM	10-fold CV (8 subjects)	Yes	11-class	81.6				
Alizadeh and Omranpour (2023)	EM-CSP + KNN	10-fold CV	No	11-class (one vs. all)	97.3				
				binary	79.2–93.5				
Hernandez-Galvan et al. (2023)	CNN+GRU	meta-training and testing	No	C/V	99.9				
				Nasal	99.9				
				Bilabial	99.9				
				iy	99.9				
				uw	99.9				
				11-class	91.5				
Current work	DE-DDA + SVM	DE-DDA + 5-fold	Yes	C/V	87	0.86	0.88	0.98	0.93
				Nasal	77	0.81	0.76	0.60	0.65
				Bilabial	79	0.82	0.76	0.63	0.68
				iy	80	0.85	0.76	0.68	0.71
				uw	93	0.87	0.73	0.41	0.51
	DE-DDA + SVM	LOO	No	C/V	82	0.47	0.99	0.82	0.90
				Nasal	65	0.49	–	0.08	–
				Bilabial	65	0.49	–	0.15	–
				iy	67	0.56	–	0.19	–
				uw	91	0.52	–	0.01	–

EM-CSP, efficient multiclass common spatial pattern; GRU, gated recurrent unit; LPCC, Linear predictive cepstral coefficients; pCBCSD, partial Coherence-Based Correntropy Spectral Density; MFCC, Mel frequency cepstral coefficients; RBM, restricted Boltzmann machine; SMRT, Sequency mapped real transform; RD-CSD, Riemannian distance of correntropy spectral density; SD, subject-dependent.

In this work, we aim to test a non-linear speech decoding method based on delay differential analysis (DDA), a signal processing tool that is increasingly being used in the analysis of iEEG (intracranial EEG) (Lainscsek et al., 2017). DDA offers a new approach that is computationally fast, robust to noise, and involves few strong features with high discriminatory power, unlike deep learning (DL) methods, which operationalize a huge array of underlying features, making a true assessment of their power and generalizability especially problematic when relying upon small benchmarking datasets. What is more, DDA leverages non-linear features of the data, which may be inaccessible to other DL methods. We evaluate the performance of DDA classification on two public imagined speech decoding datasets and compare different DDA approaches, such as training and validation between or across participants, in addition to varying window sizes.

Furthermore, the comparison of new decoding methods to previous literature is made difficult by differences in the evaluated datasets, validation methods, and performance metrics, as well as the lack of code availability. We aim to address this gap by providing a systematic review of our method relative to other past decoding work on two benchmarking databases. We provide the necessary code to reproduce our analysis, and discuss the most adequate performance metrics for future speech decoding studies.

## 2 Materials and methods

### 2.1 Datasets

The two databases analyzed in this work were selected based on the ease of access to their data online, their popularity within the speech decoding community for benchmarking purposes (Panachakel and Ramakrishnan, 2021), and the close correspondence between their stimuli sets in terms of the types of classification problems that are typically posed.

**Dataset 1: UNL-CONICET database**

The UNL-CONICET imagined speech database is an open-access dataset published by Pressel Coretto et al. (2017). This Spanish-language dataset provides two sets of stimuli that enable decoding at the word or command level in addition to smaller linguistic units: (i) six command words (up, down, left, right, backward, forward), and (ii) five vowel phonemes (/a/, /e/, /i/, /o/, /u/). The scalp EEG data consists of six channels, recorded from active electrodes F3, F4, C3, C4, P3, and P4 and mounted according to the 10-20 system. Reference and ground were placed on the left and right mastoids, respectively. Signals were sampled at 1,024 Hz and acquired with an 18-channel Grass analog amplifier model 8-18-36 and a DataTranslation analog-to-digital converter board model DT9816.

The dataset includes EEG signals from 15 participants who performed a simple imagined speech task: after 2-s intervals in which visual and auditory stimuli represented the target, participants imagined the pronunciation of the given word (4 s), with 40 trials per target stimuli. Vowels were instructed to be imagined throughout the whole 4 s. duration of the trial, while the onset for imagining command words was given by a sequence of three audible clicks. All trials were interleaved with rest intervals of 4 s duration, in which participants could blink, move, or swallow. The dataset also contains additional blocks (10 trials per target) in which participants pronounced the words/vowels, with simultaneous recording of EEG and acoustic signals. Only the former imagined speech blocks will be used in the current work.

Taken together, the stimuli in total comprise 11 classes for one decoding problem, or two problems with six (commands) and five (vowels) classes. In this work, the latter was chosen because of the difference in the command and vowel imagery blocks during the task, and because it is the most common problem investigated in the literature for this dataset.

**Dataset 2: Kara One database**

Kara One is an open-access database containing multi-modal data recorded during imagined and vocalized speech prompts (Zhao and Rudzicz, 2015). The study was conducted at the Toronto Rehabilitation Institute, where scalp EEG, face tracking, and audio recordings were collected. This database likewise provides two sets of stimuli encompassing different linguistic levels: (i) words (pat, pot, knew, gnaw), and (ii) phonemic syllables (iy, uw, piy, tiy, diy, m, n). EEG signals were recorded with a 64-channel Neuroscan Quick-cap, a SynAmps RT amplifier, and sampled at 1 kHz. A Kinect sensor recorded the audio data and facial features during the speech production stage. The multimodality (EEG, face tracking, and audio) and versatility of this dataset make it one of the most popular in the literature for speech imagery, BCI, and related fields.

EEG data were acquired from 14 participants. Each trial consisted of successive periods, including (i) rest, during which the participant clears their mind of any thoughts; (ii) prompt presentation, where the text appears onscreen and its associated auditory file is played simultaneously; (iii) articulation, during which the participant silently articulates the prompt (2 s); and (iv) imagined speech, in which the participant imagines speaking the prompt without moving (5 s); and (v) production, when the participant speaks the prompt aloud. Each of the 11 stimuli were presented in 12 trials.

The dataset allows for different classification problems. Studies utilizing this database most commonly investigate the binary assignment of phonological categories into two classes: (i) two vowel or five consonant (C/V) syllables; (ii) three bilabial (± Bilab.) or four nasal (± Nasal) phonemes; and (iii) four high-front (± iy) or four high-back (± uw) vowels. Defining all stimuli as a unique item for a decoding problem with 11 classes is also frequently undertaken.

### 2.2 Delay differential analysis

Considering the challenges involved in scalp EEG speech decoding, we propose to investigate delay differential analysis (DDA) as a method for the quick, minimally-preprocessed, and accurate classification of EEG signals in speech-decoding paradigms. DDA (Lainscsek et al., 2019a,b, 2021) is a non-linear signal processing technique based on embedding theory.

Traditional analyses are often based on spectral features and thus have hundreds of features per data segment. Recent approaches based on artificial neural networks increase the feature space even further. DDA achieves a sparse feature space by mapping data without subjective preprocessing steps, orienting to non-linear features of the data. Meaningful, if not most, brain dynamics are now assumed to be non-linear (Stam, 2005). Therefore, DDA is efficient at embedding a meaningful set of dynamics within the data in a minimal model, whereas more traditional analyses with huge feature sets often require dimensionality reduction techniques to achieve a viable number of features for decoding. As a result, DDA has several advantages: it is noise-insensitive, less prone to overfitting, and computationally fast, making it a useful tool for analyzing neural data, particularly for BCI applications that might optimally aim for real-time analysis (Lainscsek et al., 2017, 2019a,b,c, 2021).

Specifically, candidate sets of DDA models use differential and delay embeddings for detection and classification tasks in time-series data. The optimal model is determined by data type and the delays are chosen according to a specific task. The DDA model that is consistent with EEG data contains two linear one non-linear terms, with two delays and is given by:


(1) $$\begin{array}{l} \dot{u}\left(t\right)=a_{1}u\left(t-\tau_{1}\right)+a_{2}u\left(t-\tau_{2}\right)+a_{3}u{\left(t-\tau_{1}\right)}^{2}, \end{array}$$

where *u*(*t*) is the neural time series and τ_1, 2_∈ℕ are the delays (Lainscsek et al., 2017). They are adjusted for a given participant and channel in a supervised approach. While the model is invariant, it is the model's coefficients and residual, in turn, that are used as features to discriminate between classes in the decoding problem. That is, the coefficients and errors approximate the underlying dynamics of the analyzed system and may be used as features to differentiate between classes in a time series dataset.

Several methods are available within the DDA framework, which may be selected according to the demands of the classification problem. Equation (1) is referred to as single-trial DDA (ST-DDA) and is the backbone of the DDA framework. This approach has been extended to capture the overall dynamics of multiple time series simultaneously with cross-channel or cross-trial DDA (CT-DDA) (Lainscsek et al., 2019c) and causality with cross-dynamical DDA (CD-DDA) (Lainscsek et al., 2019a, 2023). A combination of ST-DDA with CT-DDA can assess dynamical ergodicity (i.e., dynamical similarity) from time series and is termed DE-DDA (Lainscsek et al., 2021). In this work, DE-DDA is used to extract features from the EEG, leveraging the spatiotemporal aspects of the data that enable discrimination between the stimuli.

DDA features were extracted from each trial in two different ways. The first approach is a single epoch after trial onset, yielding a duration of 4 s for UNL-CONICET, and 500 ms for the Kara One dataset. The second approach is a sliding window of 700 ms, with 50% overlap to increase sensitivity to transients, and a better overall feature representation. DDA coefficients are extracted from all windows, and the resulting mean and standard deviation (across windows) comprise the eight features that are used for classification for each channel. The second approach was chosen as a good trade-off between having few features and avoiding overfitting and having more features that capture the varying dynamics that might occur across imagined trials.

### 2.3 Classification and cross-validation

Two classification methods were evaluated in each dataset with the aim to assess whether DDA succeeds better at detecting common dynamics across subjects or individually. The first involves subject-dependent (SD) models trained and validated with DE-DDA, while the second is a subject-independent (SI) approach with a leave-one-out (LOO) cross-validation (CV). In the latter, the DDA delays and classification model are selected with all but one participant, and then evaluation is performed with the remaining data, and this is done successively for all participants.

After segmenting EEG data into trials, neighboring channels are grouped into triples, yielding two triples for UNL-CONICET and twenty for the Kara One dataset, from which DE-DDA coefficients are extracted. The SD method makes use of the rich spatiotemporal aspects of the DE-DDA coefficients to account for the small number of trials found in the datasets. Specifically, trial permutations that were randomized in time (see https://osf.io/d7m3n/ for technical details) are used to extract the features for selecting the model and defining the optimal DDA delay pairs [τ_1_ and τ_2_ from Equation (1), ranging from 1 to 30 delay samples] for each participant. The standardized features then serve as input to support vector machine (SVM) (Cortes and Vapnik, 1995) classifiers, set with Gaussian kernels (kernel scale set to 2), one-vs-one coding design, and applied with 5-fold CV. From all models, the best delay pairs are then ranked and the best is chosen. This SVM model resulting from the best delay pair is now tested on the time-connected trials, and the final performance of the model is evaluated. That is, non-connected trials across channel triples are used for training (more accurately, for DDA this is structure selection, see Comstock et al., 2021) and validation, and time-connected trials are used as a test set, from which performance metrics are calculated.

To assess inter-subject generalization, the SI method was performed using only time-connected trials, grouping together data from all participants and cross-validating with the LOO approach. In this way, training and validation are implemented with all but one participant. During training, the best delay pairs and SVM classifier are defined. Testing is then performed with the remaining participant added back into the data iteratively. Whether the model will generalize across subjects will rely on how specific both the DDA features and classifiers are across different participants.

Classifier scores (or the likelihood that an observation belongs to a certain class) from all channel triples are combined into a mean score that is used for classification. Accuracy values are then calculated for both datasets in order to allow our method to be compared to other speech decoding algorithms. However, for the Kara One dataset, the area under the receiving operating curve (AUC-ROC) is better used as a performance measure, since the evaluated classification problems of this dataset involve binary and very unbalanced classes.

## 3 Results

All codes and the data required to generate the figures in this work are available online https://osf.io/d7m3n/. DDA codes are written in C, and MATLAB codes are used as wrappers to run the DDA method, classification, and plotting.

**Figure 1:**
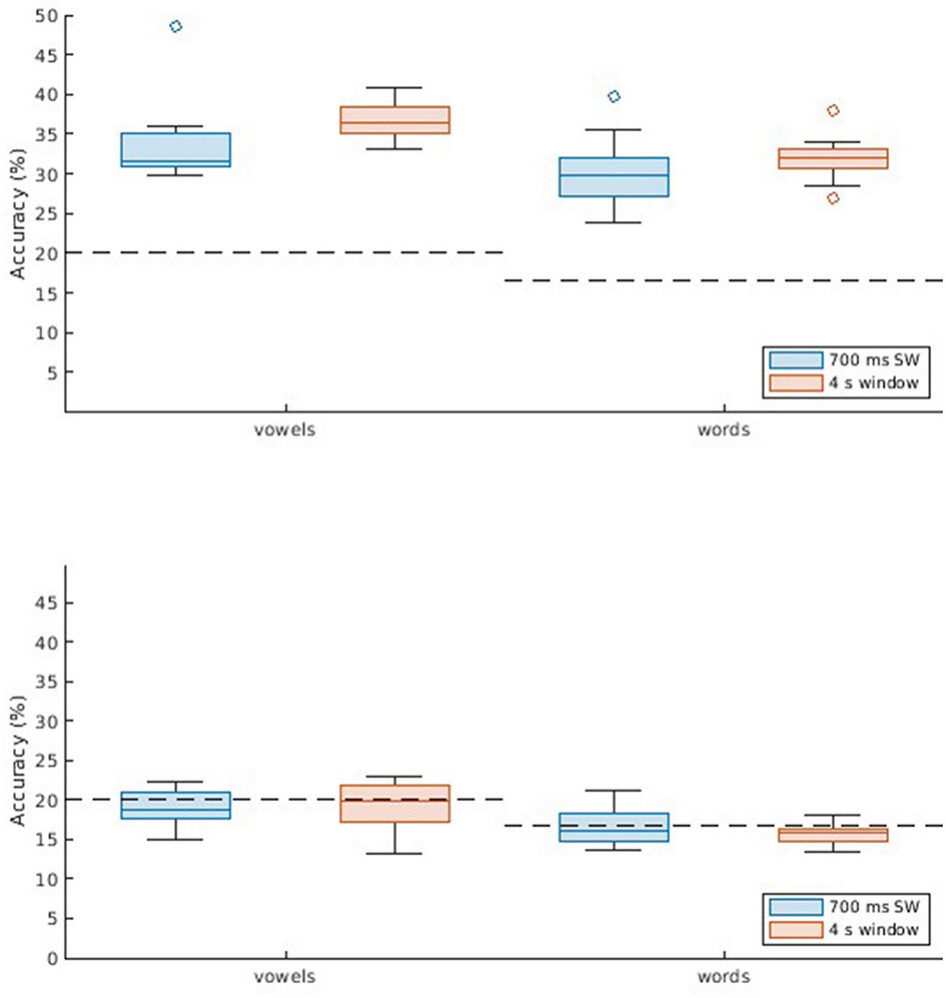
Classification results for the UNL-CONICET database. Boxplots (with median, lower/upper quartiles, and whiskers showing 1.5 interquartile range) displaying accuracy values for two classification problems: five vowels (/a/, /e/, /i/, /o/, /u/), and six command words (up, down, left, right, backward, forward). Two window lengths are assessed; 700 msec sliding window (blue), and 4 s single window (orange). Each subplot shows the results across participants for two methods: subject-dependent (SD, **Top**), using non-connected trials for training and validation and connected trials for testing; and subject-independent (SI, **Bottom**), which involves cross-subject validation.

**Figure 2:**
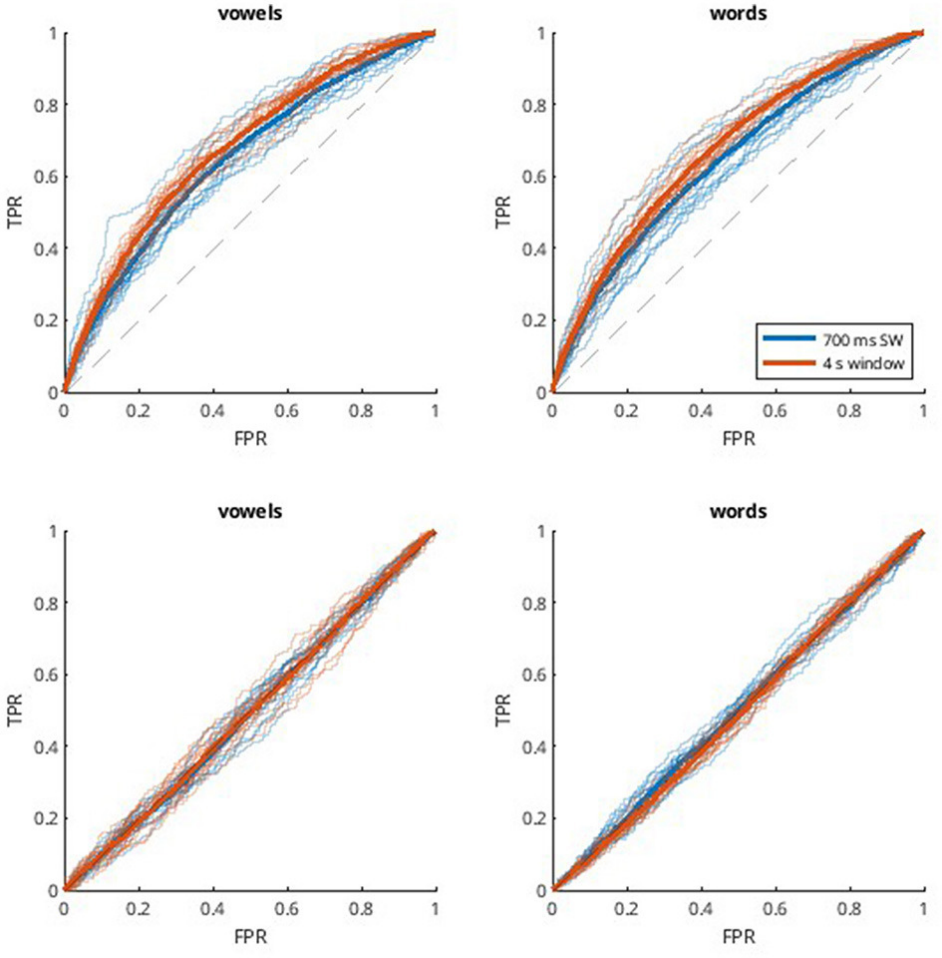
ROC curves for the UNL-CONICET database. ROC curves are obtained by considering each classification problem (vowels and words, one in each column) as different one-vs.-all binary problems, and averaging the resulting curves. Thinner and transparent lines represent each participant, and thicker ones represent the mean across participants for each case. Each subplot shows results for two window lengths; 700 ms sliding window (blue), and 4 s single window (orange). The first row shows the results for the subject-dependent method (SD, **Top**), using non-connected trials for training and validation and connected trials for testing, and the second row shows the subject-independent (SI, **Bottom**) approach, which involves cross-subject validation.

Since the UNL-CONICET dataset involves a balanced multi-class problem, performance was evaluated in terms of accuracy, as shown in Figure 1. Values are displayed for the two classification problems considered (vowels and words), and for different DDA windows; single 4-s window, and a sliding window of 700 ms. The two different classification approaches are also compared; subject-dependent (SD), and subject-independent (SI). The latter resulted in near-chance accuracies. Specifically, all SI results except the 4 s window for words (*p* = 0.008) were not significantly different from chance (20 and 16.66% for vowels and words, respectively) according to one-sample t-tests with Bonferroni correction (*alpha* = 0.0125). That is, the models are not generalizable across participants. The SD method, on the other hand, achieves accuracies of 33.6 and 36.8% for vowels, and 29.8 and 32.0% for words. For both classification problems, the single-window approach results in a slightly better performance. These differences also are observed with the receiver operating characteristic (ROC) curves in Figure 2. Computing the area under the curve (AUC) in each case, we also find a near-chance performance for the SI case (mean AUCs of 0.50 and 0.49 for 700 ms and 4 s windows, respectively), and slightly superior values for the 4 s window (0.67 for both vowels and words) compared with the sliding window approach (0.64).

**Figure 3:**
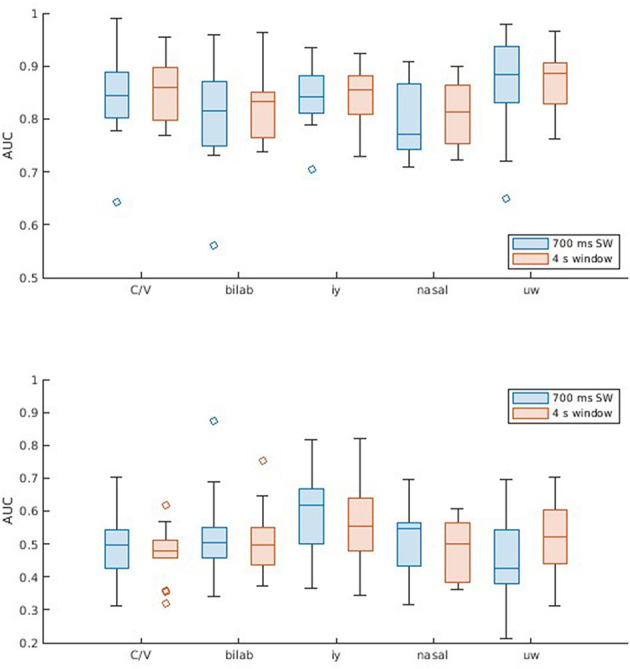
Classification results for the Kara One database. Boxplots (with median, lower/upper quartiles, and whiskers showing 1.5 interquartile range) displaying AUC values for the five classification problems (C/V, ± Bilab., ± iy, nasal, and ± uw), and different window lengths (700 ms sliding window and 4 s single window). Each subplot shows results across participants for two methods: (**Top**) SD, using non-connected trials for training and validation and connected trials for test, for each subject; and (**Bottom**) SI, which involves cross-subject LOO validation.

**Figure 4:**
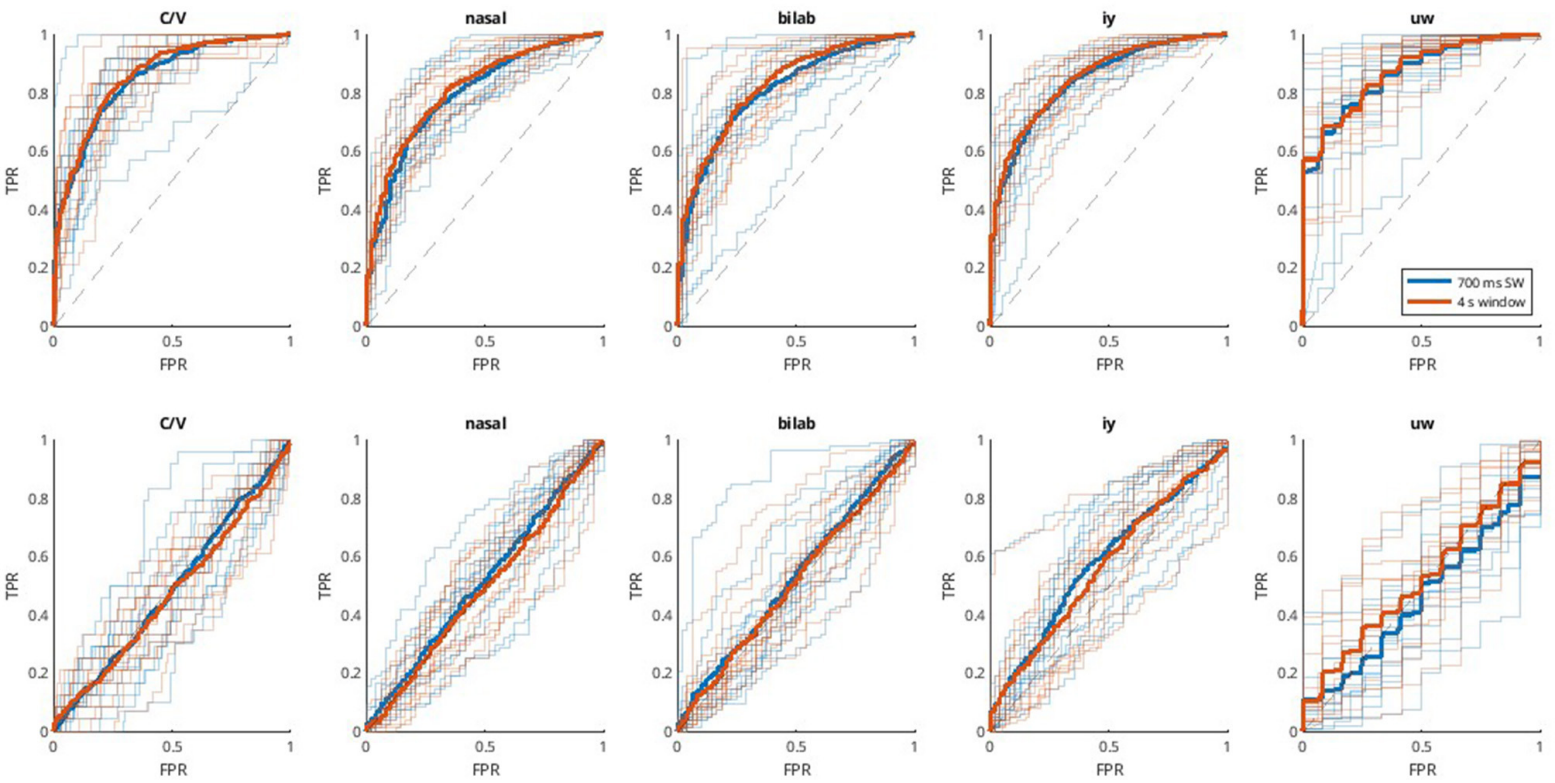
ROC curves for the Kara One database. ROC curves applied to each of the five binary classification problems (C/V, ± Bilab., ± iy, nasal, and ± uw) in each column. Thinner and transparent lines represent each participant, and thicker ones represent the mean across participants for each case. Each subplot shows results for two window lengths; 700 ms sliding window (blue), and 4 s single window (orange). The first row shows the results for the subject-dependent method (SD, **Top**), using non-connected trials for training and validation and connected trials for testing, and the second row shows the subject-independent (SI, **Bottom**) approach, which involves cross-subject validation.

Similar procedures were used for decoding with the second dataset. However, since it comprises different binary classification problems, most of which are unbalanced, the choice of DDA delays and subsequent presentation of results is made according to the AUC. Thus, AUC values are shown in Figure 3, the respective ROC curves in Figure 4, and additional metrics in Table 2. Once again, the SI approach underperforms and does not achieve significant above-chance performance levels according to one-sample t-tests with Bonferroni correction (*alpha* = 0.0125). The SD approach, on the other hand, reaches above chance-level performances, with median values (across participants) ranging from 0.81 to 0.87, depending on the window size and classification problem. Another aspect to observe is the equivalence between window sizes, where both sliding window and single-epoch approaches reached similar performance levels.

The delay pairs τ_1, 2_ have no physical meaning in the case of non-linear systems (Lainscsek and Sejnowski, 2015), but are important parameters for the DDA method. We, therefore, aimed to investigate how the best and worst delays are distributed for the different classification problems and window sizes. Toward the end of this manuscript, Section 5, we will show the delay distributions for the different classification tasks. As discussed in that section, the relation between delay pairs and classification performances have similarities across tasks and data sets.

Delay pairs were ranked according to their performance during the training stage, and mean rankings were obtained. Mean rankings were then normalized according to min-max scaling, with 0 indicating the worst-performing delay pairs for the given problem, and 1 being the best delay pairs. Considering only SD models showed satisfactory performance, only the results for the SD approach are shown here.

**Figure 5:**
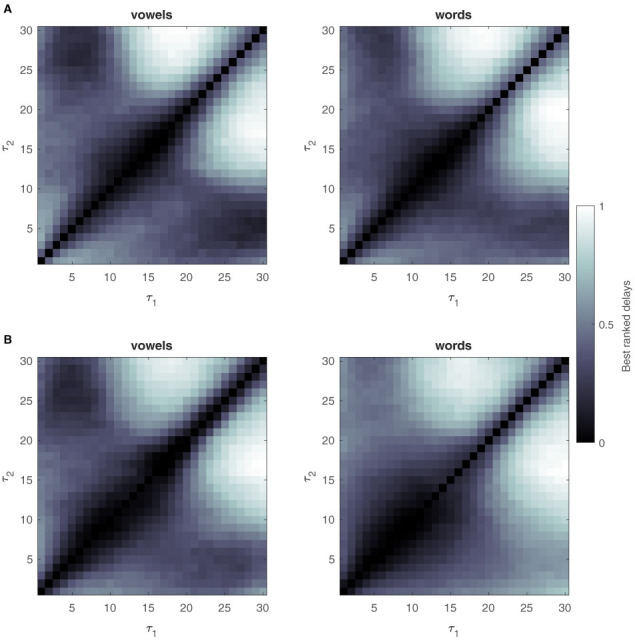
Ranking best delay pairs for the UNL-CONICET database. Delays are ranked according to their performance during the structure selection or training stage. Ranked values are averaged across participants and channel triples and min-max scaled, resulting in worst (0, black) to best (1, white) delay pair values. Results for 700 ms sliding windows are shown at the top **(A)**, for vowels and words classification problems. **(B)** Bottom plots show the same but for the 4 sec single-window approach. The exact diagonal is filled with empty values since the DDA model structure involves terms with different delays.

**Figure 6:**
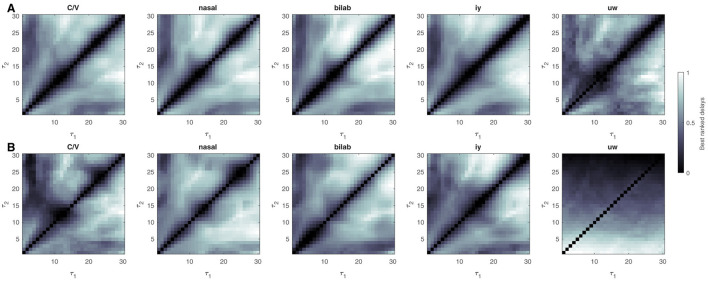
Ranking best delay pairs for the Kara One database. Delays are ranked according to their performance during the structure selection or training stage. Ranked values are averaged across participants and channel triples and min-max scaled, resulting in worst (0, black) to best (1, white) delay pair values. Results for 700 msec sliding windows are shown at the top **(A)**, for the different classification problems (C/V, ± Nasal, ± Bilabial, ± iy, ± uw). **(B)** Bottom plots show the same but for the 4 sec single-window approach. The exact diagonal is filled with empty values since the DDA model structure involves terms with different delays.

Results for the UNL-CONICET dataset are shown in Figure 5. Delay ranking distributions for the Kara One dataset are shown in Figure 6. Note, that similar delay ranking distributions are present for the different plots that represent different tasks. While the values of the delays do not have any direct connection to frequencies (see Lainscsek and Sejnowski, 2015 for a detailed analysis) the similarity of the plots shows that the DDA model captures the relevant dynamics of the system.

## 4 Comparisons

Speech decoding methods have rapidly evolved in the last few years. We review the performance of these methods and compare them to DDA for the UNL-CONICET database in Table 1 and the Kara One dataset in Table 2.

The decoding accuracies reported in the original paper on the UNL-CONICET database was only slightly above chance (Pressel Coretto et al., 2017). Spectral features were obtained by discrete wavelet transform (DWT) and combined with SVM or Random Forests (RF), yielding a mean accuracy of 22.3% for vowels and 18.6% for words. This combination of signal processing methods and traditional classifiers (such as SVMs, LDA, or RFs) in recent years has given way to more complex approaches involving deep learning architectures like CNNs and RNNs.

Cooney and colleagues (Cooney et al., 2019) employed convolutional neural networks (CNNs) with different transfer learning (TL) approaches, achieving a maximum overall accuracy of 35.7% for vowels. The same group later (Cooney et al., 2020) optimized hyperparameters for CNNs and improved on previous methods, achieving 30 and 25% for vowel and word tasks, respectively. More recently, Sarmiento and colleagues proposed another CNN approach that led to accuracies of 65.6 and 50.0% for intra- and inter-subject classification, respectively (Sarmiento et al., 2021).

A consistent finding across UNL-CONICET studies is that words, unsurprisingly, appear to pose a more complex decoding problem than isolated vowel phonemes. Although phonemes, or the phonological features offered in the Kara One dataset, might not appear to be the end goal of imagined speech decoding, they may serve as an intermediate step in the process (Saha et al., 2019), particularly given their higher decoding accuracies; they may also guide the choice of more discernible command words or stimuli for EEG-based BCIs (Comstock et al., 2019). With a few exceptions (Cooney et al., 2019; Sarmiento et al., 2021), intra-subject studies also generally outperform inter-subject analyses due to individual variabilities in the quality and features of the measured EEG signals.

Regarding studies that utilize the Kara One database, binary classification problems involving phoneme and phonological categories have more frequently been investigated. The original paper by Zhao and Rudzicz (2015) achieved between 80 and 91% accuracy for ± uw and C/V problems, respectively, using statistical and spectral features with a Deep Belief Network (DBN). Bakhshali et al. (2020) used Riemannian distance of correntropy spectral density to achieve 86 and 83% on the same problems.

One of the most consistent methods across classification problems, and the one which achieved some of the best results with this dataset is from Saha et al. (2019), who used a hierarchical combination of spatial and temporal convolutional neural network (CNN) cascaded with a deep autoencoder (DAE). The proposed approach first used this architecture to differentiate between different phonological categories (CxV, bilabial, nasal, etc.). The output latent vectors of this step are then used for classifying the 11 phonemes and words. This systematic, two-stage approach achieved an average accuracy of 83.42% across the binary phonological classification tasks, and 53.36% for the 11-class prediction task. Notably, this is one of the few studies that presents multiple evaluation metrics in addition to accuracy for the binary classification problems.

However, these results were nonetheless surpassed by combinations of more recent deep learning architectures and signal processing techniques (Mini et al., 2021; Bakhshali et al., 2022; Alizadeh and Omranpour, 2023; Hernandez-Galvan et al., 2023), as shown in Table 2. Hernandez-Galvan et al. (2023) employed a combination of convolutional and bidirectional gated recurrent unit (GRU) layers in the beta frequency band for input embedding, followed by meta-training and meta-testing, leading to a nearly perfect performance across all classification tasks, including the 11-class problem. These advancements in combining deep learning architectures and signal processing techniques have set a new standard for classification performance, prompting further exploration and refinement of these methods in imagined speech decoding. However, considering the limited signal-to-noise ratios of the involved EEG signals and the small sizes of the current datasets, it is important to examine the generalizability of these models and the underlying reasons for the significant performance improvements over traditional methods. All these deep learning methods work in a high-dimensional space of thousands of parameters compared to a sparse DDA representation as presented in this manuscript. The data in all studies mentioned here are from mostly 15 subjects and not more than 40 trials per task. Investigating the possibility of overfitting and leakage is therefore needed for methods where the number of parameters gets close to the amount of data.

As we can see, tremendous gains in decoding accuracy have been obtained for the Kara One database, whereas the most successful attempt with the UNL-CONICET database reports not much higher than chance accuracy: 66% (Sarmiento et al., 2021). As the two databases offer classification tasks that are allegedly of comparable complexity (see Cooney et al., 2022 for a review of speech decoding tasks), this phenomenon raises concerns. While the Kara One database can be considered more flexible as a data source for benchmarking studies in that it presents a large number of possible classification tasks, caution should be taken because some of the problems deal with few trials and an uneven allocation of data between classes. In these cases, the use of accuracy as a performance metric might be misleading because the model may simply be predicting the majority class. This highlights the need to transition to performance metrics, such as AUC, which accurately gauge the model's capability to differentiate between unbalanced classes by measuring its performance across various threshold settings. Overall, the DDA approach has been shown to consistently perform above chance levels, with median AUC values ranging from 0.76 to 0.9 across participants.

Our study also explored the distribution and impact of delay pairs in the DDA method when applied to the datasets, highlighting their significance in different classification scenarios and shedding light on the relationships between delay values and model performance.

## 5 Discussion

Crucial differences in the DDA approach presented here are (i) the use of a few strong features for classification, as opposed to the large-scale networks that characterize deep learning methods; and (ii) the current work provides the code for reproducing the results. Item (i) is relevant because the compact and non-linear component of DDA signal processing may add new features that are untapped in previous deep learning methods. The significance of item (ii) lies in the fact that, while deep learning models appear to perform better than the DDA model presented here, the lack of publicly available codes and proper metrics makes if difficult to evaluate if these reflect the real potential of the algorithm for generalization to new datasets. In sum, incorporating the DDA framework into other methods may very well contribute to the overall development of the field.

When investigating the distribution of best and worst delays, the main conclusion is that they are not random, but show characteristic distributions across different classification problems. This implies that there is structure in the data, and that similar dynamics might be captured by DDA across different problems, especially for the UNL-CONICET dataset. Furthermore, the low values near the main diagonals indicate that the relevant linear and non-linear components have different timescales for the investigated problems.

Refinements to the DDA method should also be considered. Here, we compared two windows to assess what data segments best serve as input to the DDA algorithm. Opposed to what we expected, the sliding-window approach did not outperform the single window one, being slightly inferior to it in the first dataset, while both are equivalent in the Kara One dataset. For the former, the task instructions for the vowels block might be related with this, since vowels were supposed to be imagined continuously throughout the whole trial, while words had auditory cues that indicated the onsets. Overall, the simplifying approaches used in this work (taking the mean classifier score across channel triples, and taking the mean and standard deviation across windows), originally aimed for simplification and avoiding overfitting issues, might have played a role in this. This highlights the challenge of dealing with the highly heterogeneous distribution of relevant dynamics throughout trials (and across channels, classes, and participants), characteristic of tasks involving imagined speech components that do not involve a stimulus-triggered response, but rather an imagined component that may be distributed heterogeneously throughout the trials. Regarding this, previous work with invasive ECoG recordings (Mugler et al., 2014) has used small window segments for the classification of all American English phonemes. Thus, a potential future direction for our work is to investigate the length and selection of optimal windows.

In both datasets, the SI approach resulted in near-chance level performance. This likely reflects the specificity of DDA delay coefficients for each participant. Another possibility is that among participants, the assumptions from DE-DDA of dynamical similarity between trials are no longer valid. This would be a considerable limitation in applying the proposed method in a subject-independent manner.

As discussed elsewhere (Sampson et al., 2019), another general limitation of the DDA framework is the difficulty in interpreting the resulting coefficients and delays, which do not map into frequency components as in the case of linear methods. Despite this, it is still possible to connect the non-linear terms and coefficients to a Volterra series (Worden et al., 1997), which can be used to determine how energy is transferred from harmonic inputs to sum and difference frequencies in the output. The extension of these ideas can be found in Zhu et al. (2022), and it could be investigated whether they are applicable to the case in this study.

Furthermore, another limitation of this study concerns the datasets used to evaluate the method. In addition to common challenges faced by imagined speech EEG datasets (low SNR, few participants and trials), it is important to remark that both datasets used in this work involved healthy participants. BCI decoding in clinical populations usually faces higher variability related to the underlying pathologies, which may affect the performance of the decoders (Lopez-Larraz et al., 2017; Lazarou et al., 2018). Thus, testing the proposed methods in patients with different degrees of speech and motor impairment is an important next step for their application in clinical settings.

## 6 Conclusion

In this study, a speech decoding method is proposed using the delay differential analysis (DDA) framework and is evaluated on two publicly available datasets involving different imagined speech classification problems: the identification of words, phonemes, and phonological features. A subject-independent application of the method did not generalize and resulted in near chance levels, whereas within-subject models performed in alignment with other classification attempts from the literature, with accuracies of 36.7% (vowels), and 33.6% (words) for the UNL-CONICET database. For the Kara One database, we report and AUCs ranging from 0.82 to 0.88 for binary phonological classification problems.

The absence of publicly available code and variations in validation methods among studies pose significant challenges in assessing and comparing speech decoding methods. We have made the code available to facilitate its integration into other frameworks and leverage the potent, compact features inherent to the DDA method. We thus aim to promote research that is reproducible, collaborative, and embraces an open-science approach.

## Data availability statement

Publicly available datasets were analyzed in this study. This data can be found here: Kara One dataset is available at: http://www.cs.toronto.edu/c~omplingweb/data/karaOne/karaOne.html. UCN-CONICET dataset is available at: http://fich.unl.edu.ar/sinc/downloads/imagined_speech/. Scripts for reproducing the figures of the article are available at https://osf.io/d7m3n/.

## Ethics statement

The studies involving humans were approved by University of Toronto and the University Health Network. The studies were conducted in accordance with the local legislation and institutional requirements. Written informed consent for participation was not required from the participants or the participants' legal guardians/next of kin in accordance with the national legislation and institutional requirements.

## Author contributions

VC: Conceptualization, Data curation, Formal analysis, Methodology, Software, Validation, Visualization, Writing – original draft, Investigation, Writing – review & editing. EM: Validation, Visualization, Writing – original draft, Conceptualization, Investigation, Methodology, Supervision, Writing – review & editing. AF: Resources, Writing – review & editing, Funding acquisition. TS: Resources, Writing – review & editing, Funding acquisition, Supervision. LC: Conceptualization, Funding acquisition, Project administration, Supervision, Writing – review & editing, Data curation, Investigation, Resources. CL: Conceptualization, Formal analysis, Methodology, Software, Validation, Visualization, Writing – review & editing, Investigation.
